# Protein Hydrolysates from Animal or Vegetal Sources Affect Morpho-Physiological Traits, Ornamental Quality, Mineral Composition, and Shelf-Life of Chrysanthemum in a Distinctive Manner

**DOI:** 10.3390/plants11172321

**Published:** 2022-09-05

**Authors:** Petronia Carillo, Antonio Pannico, Chiara Cirillo, Michele Ciriello, Giuseppe Colla, Mariateresa Cardarelli, Stefania De Pascale, Youssef Rouphael

**Affiliations:** 1 Department of Environmental, Biological and Pharmaceutical Sciences and Technologies, University of Campania “Luigi Vanvitelli”, Via Vivaldi 43, 81100 Caserta, Italy; 2 Department of Agricultural Sciences, University of Naples Federico II, 80055 Portici, Italy; 3 Department of Agriculture and Forest Sciences, University of Tuscia, 01100 Viterbo, Italy

**Keywords:** *Chrysanthemum morifolium*, amino acids, signaling molecules, physiological status, sustainable floriculture, foliar application, mode of actions, mineral composition, post-harvest

## Abstract

Protein hydrolysates (PHs) are a prominent category of plant biostimulants, mainly constituted of amino acids, oligopeptides and polypeptides, obtained by partial hydrolysis of animal or plant protein sources. Despite scientific evidence supporting the biostimulant action of PHs on vegetables, the morphological, physiological, and shelf-life performances underlying the PH action on cut flowers are still poorly explored. Accordingly, the aim of this research is to assess the effects of three commercial biostimulants, one animal PH (PH A, Hicure^®^) and two plant PHs (PH V1, Trainer^®^ and PH V2, Vegamin©), on two chrysanthemum (*Chrysanthemum morifolium*) cultivars (Pinacolada and Radost). In both cultivars, only the plant-derived PH (V1 and V2) treatments recorded significantly higher fresh plant biomass than the control (on average +18%, in both cultivars). The foliar application of the vegetal-derived PHs but not the animal one, particularly in Pinacolada, improved the status of plants, stimulating stem elongation and the apical flower diameter. In Pinacolada, applications with PH V1 resulted in a significant increase in nitrate and P concentration in leaves and Ca content in flowers compared with the control (+43%, +27%, and +28% for nitrate, P, and Ca, respectively). In Radost, PH A and PH V2 applications caused a significant reduction in nitrate concentration in both leaves and flowers compared with the control. One week after harvest, in both cultivars, PH A applications caused flower stems to wilt faster than the control. In contrast, plants treated with PH V1 revealed significantly slower flower stem senescence compared to the control. Flower wilting during vase life was correlated to a decrease in the K-to-Na ratio in flowers due to an inability to transport K to the flowers from the leaves rather than an increase in Na in the flowers themselves.

## 1. Introduction

Ornamental plants are very diversified products and the fastest growing horticultural industry [1].

Among the top-selling and most famous cut flowers in the world, with a prominent position in the cut flower industry, is *Chrysanthemum morifolium* (Asteraceae). Chrysanthemums’ big flower dimensions and very long vase life (post-harvest duration) are among the preferable quality traits that account for their great ornamental value [2,3]. Germany, France and the United Kingdom are the main markets for cut chrysanthemums in Europe, while The Netherlands, with 800 million stalks per year, and Italy, with 500 million stalks per year, are the main producers that dominate the European market [3,4]. However, cut flower production and handling are not eco-friendly processes; in fact, the farming and post-harvest handling of fresh flowers are correlated with a significant environmental and health impact. To boost chrysanthemum production in order to meet consumer demand, large inputs of materials and energy are required, both for fertilizer and pesticide production and application and, in winter or in northern countries (e.g., The Netherlands and the UK), for lightening and/or heating greenhouses [5,6]. Moreover, for prolonging cut flower vase life and delaying ethylene production and/or action and, therefore, senescence, chemical pulsing treatments, including silver thiosulfate (STS), hydroquinone (HQ), 8-hydroxyquinoline sulfate (8-HQS), silver nitrate (AgNO_3_), (aminooxy)acetic acid (AOA), calcium dichloride, cobalt chloride (CoCl_2_), aluminum sulfate (Al_2_(SO_4_)_3_), chlorine dioxide (ClO_2_), and benzyladenine (C_12_H_11_N_5_) as well as, more recently, cobalt, lanthanum, and selenium are applied [7,8]. For flowers, unlike other food crops, the use of synthetic chemicals is poorly regulated at the local level, and no maximum residue limits (MRLs) have been set [9]. Cut flowers are usually sprayed with these rather toxic chemicals and then shipped directly to markets, with no gap between processing and harvesting [9]. Therefore, the environmental and health risks associated with the massive and unregulated use of chemicals during production and vase life are particularly high for producers, florists, people who live near the production sites and, albeit to a lesser extent, for consumers. Therefore, reducing the environmental impact and health risks associated with cut flower production and post-harvest handling is of pivotal importance for the sustainable development of this sector.

A promising strategy to reduce the environmental impact and health risks associated with cut chrysanthemum flower production and post-harvest handling and to sustainably contribute to the development of this sector entails the use of naturally derived biostimulants [10,11,12,13,14]. These latter are organic and inorganic compounds or microorganisms that, when applied to plants, can enhance the growth, yield, and tolerance to the stress of plants [15]. Biostimulants may improve plants’ resource use efficiency (RUE) and agricultural sustainability by compensating for a reduction in the supply of fertilizers to the soil [16]. An *Ascophyllum nodosum* algal extract significantly affected *Chrysanthemum* sp. root dry matter, stem height, and diameter [17]. Spraying cut *Chrysanthemum morifolium* Ram. flowers with putrescine (150–300 ppm) enhanced their freshness and vase life over control by increasing relative weight and decreasing wilting percentage [18,19]. Willow bark extract and a commercial root biostimulant complex increased root branching and adventitious root formation, thus enhancing the propagation efficiency of chrysanthemum cuttings [20]. Gawade et al. [21] compared the effects of biostimulants of different origins—animal (panchgavya, a fermented product made from cow milk, urine, dung, curd, and clarified butter), algae (seaweed extracts), plant (banana pseudo-stem sap), microorganisms (*Azotobacter* sp. or phosphorus-solubilizing bacteria), and/or humic acids—on the morphometric parameters related to growth and flowering of chrysanthemum cv. Ratlam. Indeed, the use of biostimulants enhanced the earliness of flower initiation and flowering (up to 50%), increased flower stalk length and flower stalk thickness, and prolonged the flowering duration. In particular, humic acid increased the number of inflorescences, while banana extracts significantly enhanced the vase life of chrysanthemum flowers [21]. However, no chemical analyses were performed on the chrysanthemum plant tissues, and the possible reasons for the interesting physiological changes were only speculatively explained [21]. Kim et al. [12] studied the effects of a commercial plant-derived biostimulant at different doses on the adventitious rooting responses of cuttings of *Chrysanthemum indicum* L. in comparison to a commercial formulation of indole-3-butyric acid (IBA) and 1-naphthaleneacetic acid (NAA) auxin. The biostimulant, differently from auxin, enhanced fine roots, antioxidant activities, and phenolic compounds via brassinosteroid (BR)-mediated processes in chrysanthemum cuttings because it contained natural low endogenous levels of this plant phytohormone [12].

Fan et al. [22] found that enhancements in the growth and development of chrysanthemum plants sprayed with humic acid depended on an increase in chlorophyll content and fluorescence and changes in the chloroplast ultrastructure, responsible for a significant increase in photosynthetic efficiency [22]. Protein hydrolysates (PHs) are a particular category of plant biostimulants, mainly constituted of amino acids, oligopeptides, and polypeptides and obtained by partial hydrolysis of animal or plant protein sources through acid, alkaline, thermal, or enzymatic hydrolysis of animal and plant residual biomasses [15,23,24,25]. In addition, PHs contain also trace amounts of mineral elements, carbohydrates, phenols, phytohormones, and other metabolites [26,27]. Clearly, their composition depends on the animal or plant protein sources and the chemical and/or enzymatic hydrolysis processes [24,28,29]. They can be applied by root drenching or foliar spraying [26]. Animal PHs have been demonstrated to be able to increase plant tolerance to abiotic stresses (e.g., water stress, salt stress, extreme temperatures, and phytotoxicity) and improve plants’ resource use efficiency (RUE). However, since alkalis, acids, and/or high temperatures are used for their production, they may contain high levels of chloride, sodium, and other salts, and repeated or high dose use can be toxic [30]. Moreover, they are scarce in thermolabile amino acids and, in particular, minor amino acids such as tryptophan, which plays an important physiological role in plants as a precursor of auxin [31]. On the contrary, vegetal PHs have no dosing issues or salt-related toxicity because they are usually produced by partial chemical (acid and alkaline hydrolysis) or thermal hydrolysis [15,25,26] and, above all, enzymatic proteolysis at temperatures always lower than 60 °C and at pH values close to neutrality, thus preserving the thermolabile amino acids (particularly essential amino acids) [26,32]. Plant PHs may enhance plant RUE [33,34], carbon and nitrogen metabolism [26,28,29], yield and quality [28,33,35], and tolerance to abiotic stresses [29,36,37]. They contain bioactive peptides able to elicit auxin and/or gibberellin-like activities, thus increasing the density, number, length, and surface of lateral roots and, indirectly, nutrients uptake and use efficiency, plant growth, and yield [24,28,38]. Moreover, plant PHs contain peptides and amino acids (e.g., aspartate and glutamate) that are able to function as iron chelates, efficiently carrying Fe into plants, or signal molecules for the microbiota present in the rhizosphere, thus improving the Fe-nutritional status of plants [39,40,41].

With this premise, the aim of our paper is to verify the beneficial effect of commercial PHs, one of animal origin (PH A) and two of plant origin (PH V1 and PH V2), on the growth, quality parameters, and vase life of two chrysanthemum cultivars (Pinacolada and Radost) in comparison with an untreated control. The morpho-physiological data obtained from the different treatments allowed us to highlight that the PHs, particularly those of vegetal origin, were able to increase growth and quality parameters in a cultivar-specific manner. Moreover, one of the two vegetal PHs (PH V1) was able to modulate a decrease in the K-to-Na ratio in flowers during vase life, hence reducing flower wilting and senescence.

## 2. Results

### 2.1. Biomass Production and Quality Parameters

**Figure 1:**
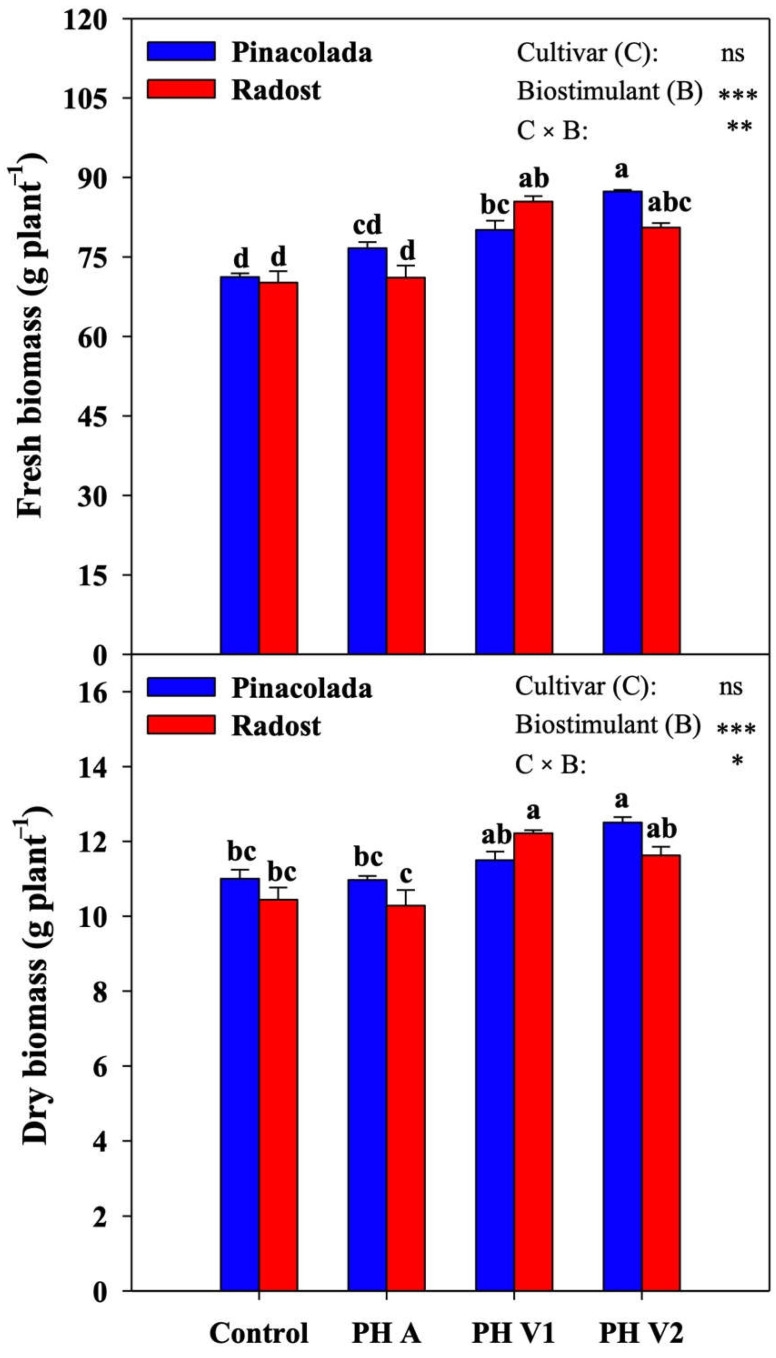
The effect of biostimulant treatments (untreated control, one animal PH (PH A, Hicure^®^) or two vegetal PHs (PH V1, Trainer^®^ and PH V2, Vegamin^®^)) on fresh and dry biomasses of two chrysanthemum cultivars (Pinacolada and Radost) grown under greenhouse conditions. ns, *, **, *** Nonsignificant or significant at *p* ≤ 0.05, 0.01, and 0.001, respectively. Different letters within each column indicate significant differences according to Tukey’s HSD test (*p* = 0.05).

In our work, fresh and dry plant biomasses were not significantly different among cultivars (Figure 1). However, fresh and dry biomasses were influenced by cultivar and biostimulant treatments, with significant C × B interaction. In both genotypes, only plant-derived PH treatments recorded significantly higher fresh plant biomass than the control (on average +18% in both cultivars). Specifically, in Pinacolada, the application of PH V2 showed a significant increase in fresh biomass compared to all other treatments (+23% compared to control) (Figure 1).

Similarly, the same cultivar recorded the highest dry plant biomass under the PH V2 treatment (+14% compared to control). Conversely, in Radost, the highest values were observed with PH V1 applications (+17% compared to control). Regardless of biostimulant treatment, Pinacolada exhibited a lower number of flowers than Radost; in contrast, the same cultivar revealed significantly greater stem length and apical flower diameter than Radost (Table 1). However, no significant difference was found in terms of stem diameter and plant dry matter on the average effect of the genotype. The average effect of the biostimulant showed a significant increase in flower number, stem diameter, and plant dry matter in all PH applications compared to control (Table 1). Stem length was found to be significantly higher only in plants treated with plant-derived biostimulants compared to control (on average +4%). Apical flower diameter was affected by cultivar and biostimulant treatments, with significant C × B interaction (Table 1). In Pinacolada, plant-derived PH treatments recorded significantly higher apical flower diameter than the control (on average +22%), while in Radost, animal-derived biostimulant applications significantly decreased the apical flower diameter compared with all other treatments (−16% compared to control).

Table 2 shows the percentage distribution of flower stems in the two main commercial categories and the unmarketable waste product. Regardless of biostimulant treatments, Pinacolada produced a significantly higher percentage of first-category flower stems than the cultivar Radost; the latter, at the same time, exhibited a higher waste percentage than Pinacolada. The average effect of biostimulants revealed a significant decrease in the percentage of unmarketable stems in all PH-treated plants compared to control (Table 2). In particular, the application of PH V1 recorded the lowest waste percentage compared to all the other treatments (3.6-fold less than control). Percentages of flower stems belonging to the first and second categories were influenced by cultivar and biostimulant treatments, with significant C × B interaction. Pinacolada treated with PH V2 showed the highest percentage of first-category stems (+30% compared to control) and the lowest percentage of second-category stems (−15% compared to control) compared to all other treatments. In Radost, applications of plant-derived biostimulants produced a significantly higher percentage of first-category stems compared to the other treatments (Table 2). The same cultivar treated with PH A recorded the highest percentage of second-category stems compared to the other treatments (+8% compared to control).

### 2.2. Physiological Measurements

The average effect of cultivar revealed a significantly higher SPAD index in Pinacolada than in Radost, while no significant difference was found in terms of chlorophyll fluorescence (Table 3). Regardless of cultivar, the plant-derived PH applications recorded significantly higher SPAD index values than the other two treatments. Chlorophyll fluorescence showed a significant interaction; specifically, the Radost cultivar treated with PH V1 exhibited significantly higher values than the plants treated with PH V2; in contrast, Pinacolada revealed no significant difference for this parameter.

### 2.3. Mineral Composition

Table 4 shows the mineral composition of the leaves and flowers of the plants of the two cultivars treated with different PH applications. Regardless of biostimulant treatments, Pinacolada exhibited, in both leaves and flowers, higher phosphorus and sulfur content but significantly lower nitrate concentration than Radost. Potassium in leaves and calcium in flowers were significantly higher in the cultivar Radost than in Pinacolada; in contrast, calcium concentration in leaves was significantly higher in Pinacolada compared to Radost (Table 4). The average effect of biostimulants revealed higher Ca and Mg content in the leaves of PH-V1-treated plants compared to control, whereas the Mg concentration in flowers was significantly higher under the PH V1 treatment compared to the PH V2 treatment. Plants treated with PH A showed significantly higher leaf K content than control; in contrast, in flowers, K concentration was lower in PH A and PH V2 treatments than in control and PH V1l. All biostimulant treatments significantly reduced the Na content in flowers compared to control; conversely, PH-A-treated plants recorded higher leaf Na concentration than control (Table 4).

**Figure 2:**
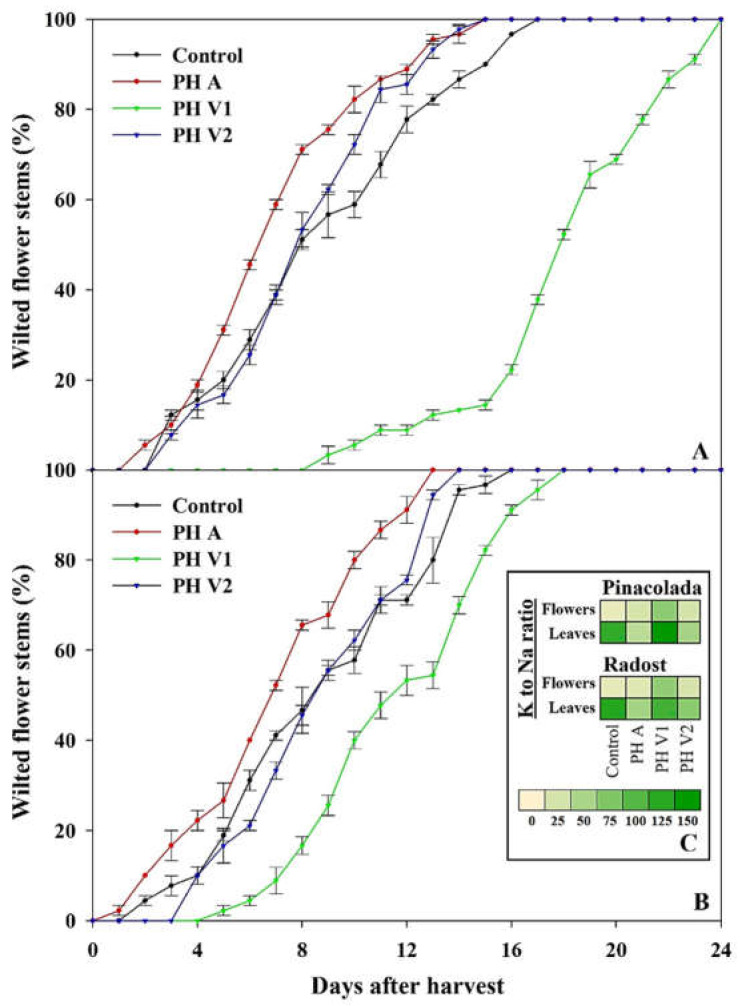
Flower stem wilting in Pinacolada (**A**) and Radost (**B**) cultivars, as affected by the biostimulant treatments (untreated control, one animal PH (PH A, Hicure^®^), or two vegetal PHs (PH V1, Trainer^®^ and PH V2, Vegamin^®^)). In the figure inset (**C**), a heat map of the K-to-Na ratio in the flowers and leaves of Pinacolada and Radost cultivars under the different treatments. The heat map was visualized using a yellow-to-green false incremental color scale.

However, the K-to-Na content was lower in flowers than in leaves in all treatments except for PH V1 (Figure 2C). Mineral composition in terms of nitrate, both in leaves and flowers; P, S, and Cl in leaves; and Ca in flowers were influenced by cultivar and biostimulant treatments, with significant C × B interaction. In Pinacolada, applications with PH V1 resulted in a significant increase in nitrate and P concentrations in leaves and Ca content in flowers compared to control (+43%, +27%, and +28% for nitrate, P, and Ca, respectively). On the same cultivar, plant-derived biostimulant applications increased leaf S content compared to control, while leaf Cl concentration was significantly lower in plants treated with PH V1 than in all other treatments (−40% compared to control). In Radost, PH A and PH V2 applications caused a significant reduction in nitrate concentration in both leaves and flowers compared to control (on average, −20% and −40% in leaves and flowers, respectively). Ca content in PH-V1-treated Radost flowers was significantly higher compared to control (+49%); in contrast, leaf Cl concentration of the same cultivar was significantly lower in all plants treated with biostimulants than in control (on average, −29%).

### 2.4. Vase Life of Flower Stems

The two cultivars exhibited a distinctive response to the application of the different biostimulants in terms of wilted flower stems (Figure 2). Specifically, one week after harvest, Pinacolada plants under control, PH A, PH V1, and PH V2 treatments displayed 38.9%, 58.9%, 0.0%, and 38.9% wilted flower stems, respectively (Figure 2A), whereas Radost plants subjected to the same treatments showed 41.1%, 52.2%, 8.9%, and 33.3% wilted flower stems, respectively (Figure 2B). In both cultivars, PH A applications caused flower stems to wilt faster than the control. In contrast, plants treated with PH V1 revealed significantly slower flower stem senescence compared to control (Figure 2). Notably, two weeks after harvest, the Pinacolada cultivar treated with PH V1 exhibited only 13.3% of wilted flower stems, while the plants of control, PH A, and PH V2 treatments displayed 86.7%, 96.7%, and 97.8% of wilted flower stems (Figure 2A). The preservability of Pinacolada flowers treated with PH V1 was not equally evident in the Radost cultivar (Figure 2).

### 2.5. Principal Component Analysis (PCA)

**Figure 3:**
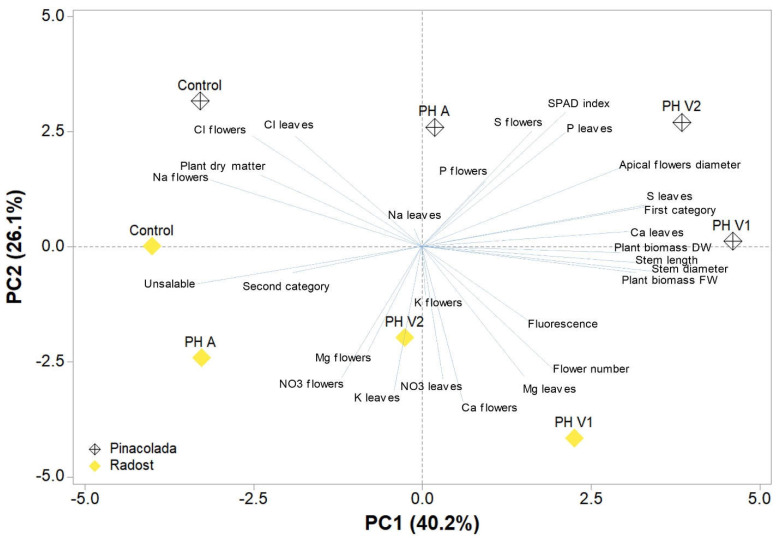
Principal component loading plot and scores of principal component analysis (PCA) of growth parameters (plant fresh and dry biomasses, flower number, stem diameter and length, apical flower diameter, plant dry matter), flower category (first, second, and unsaleable), SPAD index, chlorophyll fluorescence, and leaves and flowers’ mineral profile of two chrysanthemum cultivars (Pinacolada and Radost) under control, one animal PH (PH A, Hicure^®^), or two vegetal PHs (PH V1 and PH V2).

Principal component analysis was performed on the two chrysanthemum cultivars (Pinacolada and Radost); the analyzed data in relation to the different biostimulant applications, cultivars, and biostimulant treatments and the loading plot and scores are reported in Figure 3. The variables in the first four principal components (PCs) are highly correlated, with eigenvalues greater than 1, thus explaining 88.3% of the total variance, with PC1, PC2, PC3, and PC4 accounting for 40.2%, 26.1%, 16.3%, and 6.7%, respectively. The use of PH-based biostimulants contributed to the clear separation of PC1, whereas the distribution of PC2 was linked to the cultivars. PC1 is positively correlated with stem diameter and length, first-category flowers, leaf S and Ca contents, plant fresh and dry biomasses, and apical flower diameter and negatively correlated with unsaleable and second-category flowers, flower Na and Cl contents, and plant dry matter. In addition, PC2 is positively correlated with the SPAD index, flower S and leaf P, and Cl contents, while it is negatively correlated to the flower content of Ca, NO_3_^−^, and Mg, the leaf content of K, NO_3_^−^, and Mg, and flower number. The two chrysanthemum cultivars are well separated but not uniformly clustered with respect to PC1 and PC2. In fact, Pinacolada under different biostimulant treatments is distributed on the positive side of PC2, between the upper left and right quadrants, while Radost under different treatments is distributed on the negative side of PC2, between the lower left and half of the right quadrants. Moreover, both the control treatments and Radost PH A are distributed in the negative side of PC1, between the upper (Pinacolada) and lower (Radost) left quadrant, correlated with flower Na and Cl contents, plant dry matter, and unsaleable and second-category flowers. In contrast, on the positive side of PC1 in the upper right quadrant, there are the Pinacolada PH V1 and PH V2 treatments, correlated with stem diameter and length, first-category flowers, leaf S and Ca contents, plant fresh and dry biomasses, and apical flower diameter. In the middle of the lower-right quadrant, there is Radost PH V1, correlated with flower number, leaf Mg and flower Ca contents, and fluorescence (Figure 3).

## 3. Discussion

In 2021, the EU held first place in cut flower and ornamental potted plant sales with 31.0% of the global value of the industry [42]. In particular, the cut flower market is foreseen to further expand, thus exerting enormous pressure on the floricultural sector to ensure production at affordable prices. Clearly, one of the possible solutions is the intensification of culture, with high use of cultural inputs in concentrated spaces, but the impact on the environment and health could be serious. In fact, greenhouse floriculture is extremely resource-intensive and involves one of the highest uses of chemical fertilizers per unit of surface compared to other agricultural systems, causing high costs and a strong ecological impact. Indeed, fertilizer prices are closely linked to energy prices, in particular to the price of oil [43]. Moreover, there are costs for warming and/or lighting greenhouses, pesticides, and other innumerable synthetic chemicals used to prolong the vase life of cut flowers. Hence, these new objectives of increasing production should be achieved by adopting environmentally friendly behaviors. This could attract even more environmentally aware consumers who would be willing to buy eco-friendly products with reduced CO_2_ footprints and lower risks to health, even at higher prices.

It has already been shown that the use of natural plant biostimulants, when applied in low doses, can improve the efficiency of use of crop resources (RUE) as well as increase yields and tolerance to abiotic stress in flowering plants [10,11,12,20,21]. It is, therefore, essential to increase our knowledge on the possibility of using these biostimulants, in particular, PHs that have proved to be very effective in diverse species (including floricultural and vegetable crops) [10,11,14,16,41], to increase the RUE, yield, quality, and vase life of cut flowers. With this premise, we used three commercial PHs, one of animal origin (PH A) and two of plant origin (PH V1 and PH V2), for the cultivation of two chrysanthemum cultivars (Pinacolada and Radost) in comparison with an untreated control. The choice of chrysanthemum was not accidental since, in Royal FloraHolland, the world’s largest market for horticultural products, it was the second-best-selling cut flower in 2020 after roses, accounting for roughly 313 million euros in revenue [44].

Principal component analysis (PCA) proved to be effective for the analysis and interpretation of the morpho-physiological data obtained from the different treatments with biostimulants in the two chrysanthemum cultivars, as has been previously reported [31,41,45,46,47] (Figure 3). In fact, it provided a qualitative–quantitative estimate of the effectiveness of plant PHs in promoting yield and quality compared not only to the respective control plants but also to those cultivated with PH A (Hicure^®^). This analysis also clearly highlighted the greater responsiveness of the Pinacolada cultivar to biostimulants and to PH V1 (Trainer^®^) and PH V2 (Vegamin^®^) compared to Radost. In fact, the foliar application of vegetal-derived PHs, but not the animal one, particularly in Pinacolada, improved the status of the plants, stimulating stem elongation and apical flower diameter. This increase in stem elongation could be a result of improved macronutrient uptake by plant biostimulants, as previously reported by [25,48]. In fact, leaf S and Ca contents in the flowers increased, thus contributing to the improvement in the fresh and dry biomasses and enhancing the percentage of flowers belonging to the first category compared to untreated controls. Accordingly, PH A Hicure^®^ did not increase the apical flower diameter and other flower quality parameters, except for the flower head diameter (but only in one trial out of two in carnation (*Dianthus caryophyllus* L.) because it was unable to stimulate the beneficial macronutrients uptake [48]. Moreover, Calvo et al. [25] and Niyokuri et al. [49] also hypothesized that the stimulation of stem growth could depend on the direct uptake of amino acids contained in biostimulants promptly used for plant growth and development.

However, the most surprising and evident qualitative–quantitative result was the correlation between the second and unmarketable category of flowers, the accumulation of sodium and chloride in leaves and flowers, and the plant dry matter in both the control and Radost PH A treatments. The accumulation of these toxic ions in plant tissues can certainly have a role in the lower RUE in plants treated with PH A because of the direct interference of Na and Cl by other beneficial macronutrients, such as K or NO_3_^−^, independently of cultivar. Indeed, the biostimulants of animal origin may have the problem of higher Cl, Na, or other salt contents compared to those of V-PHs because chemical hydrolysis uses alkalis or acids [41]. Moreover, animal-derived PHs could also contain high levels of thermostable amino acids such as glycine, alanine, hydroxylysine, hydroxyproline, and proline [41]. In particular, Han et al. [50] demonstrated that exogenous glycine may inhibit root growth and indirectly decrease nitrate uptake in *Brassica campestris*. They ascribed the root growth inhibition to the increase of ethylene due to the glycine-dependent enhancement of the enzyme activities involved in its synthesis. The possible glycine-mediated increase of ethylene synthesis in animal-derived PHs could also explain the precocity of flower wilting in PH-A-treated plants (Figure 2A,B). The mild delay in wilting seen in PH-V2-treated Pinacolada plants could also be ascribed to the ability of this biostimulant (Vegamin^®^) to reduce the ethylene synthesis and its negative effects, as reported by [51].

In addition, it is interesting to make a point that in flowers from control and PH-V2-treated plants, differently from PH-V1-treated ones, premature wilting and senescence could be correlated with a strong decrease in the potassium-to-sodium ratios (Figure 2C). As reported by Cirillo et al. [52], the reduction of the K-to-Na ratio is a parameter far more important than the absolute amount of Na because exceeding K, Na can substitute it in key enzymatic reactions, damaging cytosol and organelle functions. However, the decrease in K- to -Na ratios seems to depend more on the incapacity of translocating K from leaves to flowers than on an absolute strong increase of Na in flowers, which is able to depolarize and damage the plasma membrane and actively restrict K uptake [53]. Instead, PH V1 plants were more able to restrict Na accumulation in leaves and its transport to flowers rather than highly increasing the transport of K to flowers, thus indirectly increasing the flower K-to-Na ratio. Indeed, PH V1 (Trainer^®^) has already been demonstrated to enhance RUE in horticultural crop production [24,33,34], C and N metabolism [26,28], productivity and quality [24,33], and tolerance to abiotic stresses [27,37]; these beneficial effects been attributed to bioactive peptides with hormone-like activities (auxin and gibberellins) that are able to directly enhance root and shoot growth and indirectly promote beneficial nutrient uptake and crop yield [24,28,33,54]. Accordingly, in PH V1 plants, nitrate, Ca, Mg, and S significantly increased in leaves and flowers, while Na was reduced in flowers and Cl in leaves. Although there are no specific studies linking nitrogen deficiency or sufficiency to ethylene synthesis and senescence in flowers, Druege [55] found that N deficiency increases ethylene biosynthesis and tissue sensitivity. N deficiency during pre-harvest could affect the photosynthetic activity of plants and the life of both growing and cut flowers. Indeed, in conditions of nutrient deficiency, plants will accelerate all physiological processes related to species dispersal: these usually include the induction of flowering and the synthesis of ethylene to ensure the spread of the species [56]. Indeed this effect could be related to ion imbalance and, specifically, to the decrease of K-to-Na ratios, which can be the prodromic signal for an auxin-dependent stimulation of ethylene synthesis through an inductive action on the expression of the key enzyme ACS [57]. In fact, the increase of Na on K can exert a negative effect on the expression of the auxin carrier PIN-FORMED 2 (PIN2), avoiding its transport/efflux and, therefore, increasing its cellular content [58]. Moreover, the increase of Na on K can also activate mechanisms to prevent the accumulation of toxic Na in the cytosol, such as active Na efflux into the apoplast and its compartmentalization into the vacuole by secondary active Na^+^/H^+^ antiporters present in the plasma membrane and tonoplast [59]. The latter could change the membrane potential, altering the functioning of aquaporins and/or downregulating their expression, thus decreasing the passage of water through biological membranes, the motor cell dynamics, and, therefore, cell physiological functions, accelerating water stress and wilting [57,60].

## 4. Materials and Methods

### 4.1. Experimental Site, Plant Material, and Growth Conditions

The experiment was carried out in a heated greenhouse during the winter–spring 2020 season at the farm “Società Agricola Gargiulo & C.s.s.”, located in Torre del Greco (Naples, South Italy, latitude 40°46′ N, longitude 14°23′ E, altitude 60 m). Healthy and rooted terminal cuttings of two chrysanthemum cultivars (*Chrysanthemum morifolium*, cv. “Pinacolada” and “Radost”) were planted on raised beds at a spacing of 15 × 15 cm. During the growing cycle, the management of irrigation, soil fertility, and pest and disease control followed ordinary local practices.

### 4.2. Biostimulant Treatments and Experimental Design

Experimental blocks of 3 m^2^ each were subjected to four treatments that included a control (sprayed with water only) and three commercial biostimulants applied at the recommended dose, as follows: PH A (animal-derived protein hydrolysate, 3 mL L^−1^); PH V1 and PH V2 (plant−derived protein hydrolysates, 4 mL L^−1^). The PH A Hicure^®^ (Syngenta AG, Basel, Switzerland) used contained a balanced mixture of free amino acids and peptides (hydrolyzed proteins) of animal origin: amino acids and peptides (62.5%), total nitrogen (10.9%), and organic carbon (29.4%) [48]. PH V1 Trainer^®^ and Vegamin^®^, commercialized by Hello Nature Inc. (Anderson, IN, USA) (former Italpollina, Rivoli Veronese, Italy), consisted mainly of a mixture of amino acids (e.g., aspartic acid, glutamic acid, and essential amino acids), oligopeptides, and polypeptides [32,61]. Applications were performed every 10 days, spraying the whole plant in the early morning from transplanting until harvest. A randomized complete block design was applied in the present experiment, with treatments replicated three times (24 blocks in total); each experimental unit consisted of about a 2 m^2^ block area (80 plants).

### 4.3. Biomass Production and Quality Parameters

All treatments reached commercial flower maturity at the same time; plants of both cultivars were harvested 98 days after transplanting by cutting the stems 4 cm above ground level; 80 plants per block (240 per treatment) were subjected to the following biometric measurements: fresh plant biomass, stem diameter and length, number of flowers, and apical flower diameter. Prior to destructive analysis, all plants harvested were classified into the two commercial categories (first and second) and into the unmarketable product according to the standard criteria of the Italian flower market concerning: number of well-developed flowers per plant, number of flowers in anthesis, stem diameter and length, and absence of defects. Plant dry matter content was determined as a percentage of fresh mass to dry plant biomass after drying to constant weight in a forced-air oven at 70 °C for 72 h.

### 4.4. Soil Plant Analysis Development (SPAD) Index and Maximum Quantum Efficiency of Photosystem II Measurements (F_v_/F_m_)

Before flower stems were harvested, SPAD index measurements were carried out on fully expanded young leaves with a portable chlorophyll meter (SPAD-502, Minolta Camera Co., Ltd., Osaka, Japan). A single average SPAD value for each replicate was obtained by measuring twenty leaves per block. On the same date, chlorophyll fluorescence measurements with a portable fluorometer (F_v_/F_m_ Meter, Opti-Sciences Inc., Hudson, NH, USA) were performed on leaves adapted in the dark for 10 min. For each replicate, an average chlorophyll fluorescence value was obtained by measuring ten leaves per block.

### 4.5. Mineral Content Analysis

Dried tissues from flowers and leaves of plants belonging to the first commercial category were analyzed by ion chromatography (Dionex ICS-3000, Thermo Fisher Scientific, Waltham, MA, USA) with an IONPAC-ATC1 anion trap column (Dionex, Sunnyvale, CA, USA), an IONPAC-AG11 guard column (Dionex), and an analytical IONPAC-AS11 4-mm column (Dionex) fitted with an ASRSII 4-mm suppressor for anions (Dionex), an IONPAC-CTC cation trap column (Dionex), an IONPAC-CG12A guard column (Dionex) and an analytical IONPAC-CS12A 4-mm column (Dionex) fitted with a CSRS 4-mm suppressor for cations (Dionex), coupled to a CD20 conductivity detector (Dionex) [62,63]. The mobile phases were prepared with MilliQ water, to which, after bubbling helium to eliminate CO_2_, the solvent was added. For the analysis of anions, sodium hydroxide (NaOH) was used as the eluent: 0.5 mM from −15 min to 0 min for equilibrating the column, 1 mM from 0 to 5 min, and 1 to 15 mM from 5 to 15 min for anions elution, and finally 25 mM from 16 to 25 min for cleaning the column. For the analysis of cations, methanesulfonic acid (MSA) 20 mM was used for equilibrating (15 min), eluting (15 min), and cleaning (5 min) the column. The flow rate of the mobile phases was maintained at 2 mL min^−1^ with a Dionex GP50 quaternary pump. Minerals were identified and quantified by comparing retention time and peak area data with those of the specific reference standards (Sigma-Aldrich, St. Louis, MO, USA). Each sample, derived from the plants of each block, was analyzed in triplicate.

### 4.6. Post-Harvest Measurements

On the day of harvest, a sample of forty plants from each block (three replicates per treatment) belonging to the first commercial category was placed in tap-water-filled bins and stored in a climatic chamber at 5 °C. At daily intervals, flower stems showing wilting symptoms were counted and removed from each bin. The wilting status of the plants was assessed by measuring the downward bending angle of the flower heads or if the yellowing or rotting of the leaves or petals was observed. Vase life was determined by calculating every day the percentage of wilted flower stems out of the total number of plants placed in each bin at the beginning of the post-harvest test.

### 4.7. Statistical Analysis

All data were subjected to bifactorial analysis of variance (two-way ANOVA), Cultivar (C) × Biostimulant (B), using a general linear model and the SPSS software package (SPSS version 22, Chicago, IL, USA). The average effect of cultivar was compared according to Student’s *t*-test. The average effects and interaction of biostimulant factors were separated according to Tukey’s HSD test (*p* = 0.05). Principal component analysis (PCA) was performed on morpho-physiological and qualitative parameters and mineral contents to highlight the dominant parameters that mainly discriminated among the two cultivars under the different PH biostimulant applications using Minitab^®^ 18 statistical software (Minitab LLC, State College, PA, USA) [46]. The heat map results were calculated as Na-to-K ratios under the different biostimulant treatments and visualized using a yellow-to-green false incremental color scale.

## 5. Conclusions

Natural origin biostimulants are currently one of the most eco-sustainable strategies to increase the yield and quality of vegetable crops. Our study demonstrates that these biostimulants can also be used profitably to increase the RUE, yield, and vase life of cut flower plants such as chrysanthemum. The PHs, in particular those of vegetal origin, were able to increase the growth and quality parameters of treated plants, particularly Pinacolada, demonstrating that the effect was certainly cultivar-specific. Furthermore, the study found that flower wilting during vase life was correlated to a decrease in the K-to-Na ratio in flowers due to an inability to transport K to the flowers from the leaves rather than an increase in Na in the flowers themselves. It is interesting to note how, to aggravate this phenomenon, the possible accumulation of glycine, a thermostable amino acid in PH A, could contribute to accelerating the production of ethylene in the flowers, thus further worsening the phenomenon of wilting and senescence. While the partial inhibition of the synthesis of ethylene in PH V2 has already been reported in other works and is probably also present in PH V1, the ability to restrict the transport of Na to the flowers of PH V1 may instead contribute to increasing the vase life of flowers. Therefore, this study has been very useful in evaluating the effects of the different biostimulants and pinpointing the more effective ones for designing new and compelling protocols for floriculture that can be translated directly to the field. In addition, it also highlights some crucial phenomena related to the wilting and senescence mechanisms of potted flowers, pivotal for future studies on this important topic.

## Figures and Tables

**Table 1:** The effect of biostimulant treatments (untreated control, one animal PH (PH A, Hicure^®^), or two vegetal PHs (PH V1, Trainer^®^ and PH V2, Vegamin^®^)) on ornamental quality parameters of two chrysanthemum cultivars (Pinacolada and Radost) grown under greenhouse conditions. All data are expressed as mean ± standard error; *n* = 3.

Source of Variance	Flower Number	Stem Diameter	Stem Length	Apical Flowers Diameter	Plant Dry Matter
	(no. plant^−^^1^)	(cm)	(cm)	(cm)	(%)
Cultivar (C)					
Pinacolada	10.52 ± 0.32	0.64 ± 0.02	92.90 ± 0.88	6.73 ± 0.19	14.61 ± 0.16
Radost	11.46 ± 0.48	0.61 ± 0.01	91.36 ± 0.91	5.57 ± 0.16	14.52 ± 0.08
*t*−test	*	ns	**	***	ns
Biostimulant (B)					
Control	9.15 ± 0.19 b	0.57 ± 0.01 c	90.42 ± 0.14 c	5.78 ± 0.11 b	15.16 ± 0.15 a
PH A	11.00 ± 0.59 a	0.61 ± 0.01 b	89.26 ± 0.87 bc	5.68 ± 0.44 b	14.38 ± 0.10 b
PH V1	12.00 ± 0.40 a	0.66 ± 0.01 a	95.71 ± 1.04 a	6.76 ± 0.30 a	14.33 ± 0.08 b
PH V2	11.80 ± 0.26 a	0.65 ± 0.02 ab	93.14 ± 0.79 ab	6.37 ± 0.30 a	14.37 ± 0.12 b
	***	***	***	***	***
C × B					
Pinacolada × Control	9.07 ± 0.19	0.56 ± 0.03	90.43 ± 0.23	5.90 ± 0.07 bc	15.45 ± 0.18
Pinacolada × PH A	10.13 ± 0.19	0.62 ± 0.02	90.96 ± 0.69	6.60 ± 0.32 ab	14.31 ± 0.17
Pinacolada × PH V1	11.33 ± 0.37	0.68 ± 0.01	96.42 ± 1.60	7.41 ± 0.14 a	14.35 ± 0.09
Pinacolada × PH V2	11.53 ± 0.30	0.68 ± 0.02	93.80 ± 1.50	7.00 ± 0.17 a	14.32 ± 0.21
Radost × Control	9.23 ± 0.37	0.57 ± 0.01	90.41 ± 0.23	5.67 ± 0.22 c	14.88 ± 0.01
Radost × PH A	11.87 ± 0.98	0.61 ± 0.01	87.56 ± 0.64	4.76 ± 0.11 d	14.45 ± 0.13
Radost × PH V1	12.67 ± 0.47	0.65 ± 0.02	95.00 ± 1.52	6.10 ± 0.09 bc	14.30 ± 0.14
Radost × PH V2	12.07 ± 0.44	0.62 ± 0.01	92.47 ± 0.68	5.73 ± 0.08 c	14.43 ± 0.16
	ns	ns	ns	**	ns

ns, *, **, *** Nonsignificant or significant at *p* ≤ 0.05, 0.01, and 0.001, respectively. Different letters within each column indicate significant differences according to Tukey’s HSD test (*p* = 0.05).

**Table 2:** The effect of biostimulant treatments (untreated control, one animal PH (PH A, Hicure^®^), or two vegetal PHs (PH V1, Trainer^®^ and PH V2, Vegamin^®^)) on flower categories distribution of two chrysanthemum cultivars (Pinacolada and Radost) under greenhouse conditions. All data are expressed as mean ± standard error; *n* = 3.

Source of Variance	First Category	Second Category	Unmarketable
	(%)	(%)	(%)
Cultivar ©			
Pinacolada	72.76 ± 3.34	18.83 ± 1.79	8.42 ± 2.01
Radost	60.84 ± 2.58	19.86 ± 1.40	19.30 ± 2.07
*t*-test	***	ns	***
Biostimulant (B)			
Control	54.55 ± 1.58 d	21.58 ± 1.30 ab	23.87 ± 2.45 a
PH A	61.58 ± 4.22 c	24.11 ± 1.23 a	14.30 ± 3.26 b
PH V1	73.16 ± 1.79 b	20.18 ± 0.74 b	6.66 ± 1.68 d
PH V2	77.89 ± 3.89 a	11.50 ± 1.10 c	10.61 ± 2.88 c
	***	***	***
C × B			
Pinacolada × Control	56.64 ± 2.67 d	24.36 ± 0.82 ab	19.00 ± 2.22
Pinacolada × PH A	70.99 ± 0.45 bc	21.72 ± 1.10 bc	7.29 ± 1.49
Pinacolada × PH V1	76.90 ± 1.32 b	20.09 ± 1.20 c	3.01 ± 0.12
Pinacolada × PH V2	86.51 ± 1.11 a	9.13 ± 0.37 e	4.36 ± 1.48
Radost × Control	52.47 ± 0.97 d	18.81 ± 0.31 c	28.73 ± 1.16
Radost × PH A	52.18 ± 0.52 d	26.51 ± 0.83 a	21.31 ± 1.33
Radost × PH V1	69.43 ± 0.63 c	20.27 ± 1.13 bc	10.31 ± 0.89
Radost × PH V2	69.27 ± 0.43 c	13.87 ± 0.55 d	16.87 ± 0.37
	***	***	ns

ns, *** Nonsignificant or significant at *p* ≤ 0.001, respectively. Different letters within each column indicate significant differences according to Tukey’s HSD test (*p* = 0.05).

**Table 3:** The effect of biostimulant treatments (untreated control, one animal PH (PH A, Hicure^®^), or two vegetal PHs (PH V1, Trainer^®^ and PH V2; Vegamin^®^)) on the SPAD index and chlorophyll fluorescence of two chrysanthemum cultivars (Pinacolada and Radost) grown under greenhouse conditions. All data are expressed as mean ± standard error; *n* = 3.

Source of Variance	SPAD Index	Chlorophyll Fluorescence
		(F_v_/F_m_)
Cultivar (C)		
Pinacolada	54.99 ± 0.47	0.825 ± 0.002
Radost	43.00 ± 0.33	0.828 ± 0.003
*t*-test	***	ns
Biostimulant (B)		
Control	47.63 ± 2.48 b	0.822 ± 0.003 b
PH A	48.18 ± 2.79 b	0.826 ± 0.004 ab
PH V1	50.39 ± 2.62 a	0.834 ± 0.002 a
PH V2	49.80 ± 2.89 a	0.824 ± 0.004 b
	***	*
C × B		
Pinacolada × Control	53.09 ± 0.86 b	0.817 ± 0.001 bc
Pinacolada × PH A	54.41 ± 0.40 ab	0.820 ± 0.004 bc
Pinacolada × PH V1	56.24 ± 0.42 a	0.831 ± 0.003 abc
Pinacolada × PH V2	56.24 ± 0.51 a	0.832 ± 0.002 ab
Radost × Control	42.16 ± 0.07 d	0.827 ± 0.003 abc
Radost × PH A	41.95 ± 0.19 d	0.831 ± 0.004 abc
Radost × PH V1	44.55 ± 0.28 c	0.836 ± 0.003 a
Radost × PH V2	43.36 ± 0.35 cd	0.816 ± 0.003 c
	ns	**

ns, *, **, *** Nonsignificant or significant at *p* ≤ 0.05, 0.01, and 0.001, respectively. Different letters within each column indicate significant differences according to Tukey’s HSD test (*p* = 0.05).

**Table 4:** The effect of biostimulant treatments (untreated control, one animal PH (PH A, Hicure^®^), or two vegetal PHs (PH V1; Trainer^®^ and PH V2; Vegamin^®^)) on the mineral composition of two chrysanthemum cultivars (Pinacolada and Radost) grown under greenhouse conditions. All data are expressed as mean ± standard error; *n* = 3.

Source of Variance	Nitrate		P		K		Ca		Mg		S		Na		Cl	
	(g kg^−1^ DW)		(g kg^−1^ DW)		(g kg^−1^ DW)		(g kg^−1^ DW)		(g kg^−1^ DW)		(g kg^−1^ DW)		(g kg^−1^ DW)		(g kg^−1^ DW)	
	Leaves	Flowers	Leaves	Flowers	Leaves	Flowers	Leaves	Flowers	Leaves	Flowers	Leaves	Flowers	Leaves	Flowers	Leaves	Flowers
Cultivar (C)																
Pinacolada	35.37 ± 1.75	1.12 ± 0.06	1.35 ± 0.04	2.08 ± 0.11	60.68 ± 1.67	17.25 ± 0.89	11.00 ± 0.44	0.42 ± 0.02	4.51 ± 0.13	0.77 ± 0.04	0.35 ± 0.02	0.41 ± 0.01	0.80 ± 0.19	0.62 ± 0.12	18.58 ± 1.34	4.82 ± 0.19
Radost	45.42 ± 1.85	2.70 ± 0.23	1.01 ± 0.04	1.76 ± 0.12	69.52 ± 1.09	18.18 ± 1.12	9.63 ± 0.38	0.53 ± 0.03	4.78 ± 0.13	0.87 ± 0.04	0.28 ± 0.02	0.28 ± 0.01	0.77 ± 0.10	0.68 ± 0.11	17.16 ± 1.04	4.63 ± 0.19
*t*-test	***	***	***	*	***	ns	**	***	ns	ns	*	***	ns	ns	ns	ns
Biostimulant (B)																
Control	40.42 ± 4.23 b	2.27 ± 0.49 a	1.13 ± 0.05	2.09 ± 0.13	60.47 ± 2.48 b	19.65 ± 0.63 a	9.57 ± 0.34 b	0.42 ± 0.02 b	4.19 ± 0.07 b	0.85 ± 0.06 ab	0.26 ± 0.01 b	0.35 ± 0.03 ab	0.42 ± 0.02 b	1.16 ± 0.14 a	20.75 ± 0.77 a	5.44 ± 0.23 a
PH A	35.47 ± 1.29 c	1.63 ± 0.34 b	1.20 ± 0.11	1.75 ± 0.25	68.59 ± 1.59 a	15.55 ± 1.15 b	9.13 ± 0.54 b	0.45 ± 0.02 b	4.71 ± 0.20 ab	0.80 ± 0.04 ab	0.27 ± 0.03 b	0.33 ± 0.04 b	1.32 ± 0.27 a	0.63 ± 0.03 b	20.22 ± 1.60 a	4.83 ± 0.22 ab
PH V1	48.57 ± 2.05 a	2.36 ± 0.44 a	1.27 ± 0.10	2.16 ± 0.14	66.69 ± 2.53 ab	21.04 ± 0.34 a	12.10 ± 0.60 a	0.59 ± 0.04 a	4.98 ± 0.08 a	0.94 ± 0.04 a	0.37 ± 0.02 a	0.39 ± 0.03 a	0.47 ± 0.05 b	0.28 ± 0.03 b	12.45 ± 0.75 b	4.07 ± 0.01 c
PH V2	37.13 ± 2.14 bc	1.38 ± 0.23 b	1.13 ± 0.11	1.67 ± 0.10	64.64 ± 3.28 ab	14.63 ± 1.24 b	10.45 ± 0.30 b	0.43 ± 0.04 b	4.71 ± 0.22 ab	0.69 ± 0.06 b	0.34 ± 0.03 a	0.30 ± 0.03 b	0.94 ± 0.10 ab	0.52 ± 0.09 b	18.07 ± 0.85 a	4.56 ± 0.20 bc
	***	***	ns	ns	*	***	***	***	*	*	***	***	**	***	***	***
C × B																
Pinacolada × Control	31.15 ± 1.73 f	1.18 ± 0.02 cd	1.15 ± 0.09 bcd	2.20 ± 0.14	56.36 ± 3.47	18.26 ± 0.10	9.99 ± 0.25	0.40 ± 0.02 cd	4.13 ± 0.14	0.90 ± 0.06	0.24 ± 0.01 bc	0.41 ± 0.03	0.41 ± 0.04	1.17 ± 0.26	19.53 ± 1.15 ab	5.44 ± 0.27
Pinacolada × PH A	33.39 ± 1.91 ef	1.02 ± 0.11 cd	1.43 ± 0.02 ab	2.15 ± 0.28	67.45 ± 2.77	16.66 ± 0.23	9.94 ± 0.45	0.42 ± 0.03 bcd	4.54 ± 0.35	0.75 ± 0.03	0.33 ± 0.02 ab	0.42 ± 0.01	1.48 ± 0.54	0.59 ± 0.06	23.66 ± 0.44 a	5.23 ± 0.26
Pinacolada × PH V1	44.51 ± 1.88 bc	1.38 ± 0.05 bcd	1.46 ± 0.05 a	2.17 ± 0.31	61.29 ± 1.11	20.68 ± 0.51	13.17 ± 0.74	0.51 ± 0.01 b	5.02 ± 0.15	0.87 ± 0.03	0.41 ± 0.02 a	0.46 ± 0.00	0.36 ± 0.02	0.27 ± 0.05	11.77 ± 1.08 d	4.07 ± 0.01
Pinacolada × PH V2	32.45 ± 0.54 ef	0.92 ± 0.03 d	1.37 ± 0.05 abc	1.78 ± 0.17	57.61 ± 1.74	13.38 ± 1.72	10.88 ± 0.28	0.34 ± 0.02 d	4.36 ± 0.11	0.57 ± 0.06	0.40 ± 0.01 a	0.36 ± 0.02	0.96 ± 0.20	0.45 ± 0.02	19.38 ± 0.53 ab	4.53 ± 0.27
Radost × Control	49.70 ± 0.76 ab	3.37 ± 0.03 a	1.10 ± 0.06 cd	1.98 ± 0.22	64.58 ± 1.39	21.03 ± 0.27	9.15 ± 0.60	0.45 ± 0.03 bcd	4.24 ± 0.07	0.81 ± 0.11	0.28 ± 0.01 bc	0.30 ± 0.03	0.44 ± 0.03	1.16 ± 0.17	21.98 ± 0.40 a	5.43 ± 0.44
Radost × PH A	37.55 ± 0.57 de	2.25 ± 0.44 b	0.98 ± 0.07 d	1.35 ± 0.25	69.73 ± 1.91	14.43 ± 2.31	8.32 ± 0.76	0.48 ± 0.04 bc	4.88 ± 0.23	0.86 ± 0.06	0.22 ± 0.05 c	0.25 ± 0.01	1.16 ± 0.21	0.67 ± 0.02	16.79 ± 0.93 bc	4.42 ± 0.10
Radost × PH V1	52.63 ± 1.00 a	3.35 ± 0.10 a	1.07 ± 0.08 d	2.15 ± 0.09	72.09 ± 1.28	21.39 ± 0.44	11.02 ± 0.24	0.67 ± 0.01 a	4.93 ± 0.10	1.01 ± 0.04	0.32 ± 0.00 ab	0.32 ± 0.02	0.57 ± 0.02	0.29 ± 0.03	13.13 ± 1.08 cd	4.07 ± 0.01
Radost × PH V2	41.81 ± 0.86 cd	1.83 ± 0.23 bc	0.89 ± 0.03 d	1.56 ± 0.11	71.67 ± 1.12	15.89 ± 1.79	10.03 ± 0.44	0.51 ± 0.03 b	5.07 ± 0.33	0.80 ± 0.06	0.29 ± 0.01 bc	0.25 ± 0.01	0.92 ± 0.10	0.58 ± 0.18	16.76 ± 1.28 bc	4.58 ± 0.35
	***	*	**	ns	ns	ns	ns	*	ns	ns	*	ns	ns	ns	***	ns

ns, *, **, *** Nonsignificant or significant at *p* ≤ 0.05, 0.01, and 0.001, respectively. Different letters within each column indicate significant differences according to Tukey’s HSD test (*p* = 0.05).

## Data Availability

The datasets generated for this study are available upon request to the corresponding author.
